# Harnessing the Potential of Antibacterial and Antibiofilm Phytochemicals in the Combat Against Superbugs: A One Health Perspective

**DOI:** 10.3390/antibiotics14070692

**Published:** 2025-07-09

**Authors:** Suma Sarojini, Saranya Jayaram, Sandhya Kalathilparambil Santhosh, Pragyan Priyadarshini, Manikantan Pappuswamy, Balamuralikrishnan Balasubramanian

**Affiliations:** 1 Department of Life Sciences, CHRIST (Deemed to be University), Bangalore 560029, Karnataka, India; 2 Department of Biotechnology, Mount Carmel College, Autonomous, Bangalore 560001, Karnataka, India; 3 Department of Food Science and Biotechnology, College of Life Science, Sejong University, Seoul 05006, Republic of Korea

**Keywords:** superbugs, antibiofilm, antibacterial, quorum sensing, phytochemicals

## Abstract

The war between humans and bacteria started centuries ago. With the advent of antibiotics, there was a temporary ceasefire in this war, but the scenario soon started becoming worse with the emergence of drug-resistant strains within years of the deployment of antibiotics in the market. With the surge in the misuse of antibiotics, there was a drastic increase in the number of multidrug-resistant (MDR) and extensively drug-resistant bacterial strains, even to antibiotics like Methicillin and vancomycin, aggravating the healthcare scenario. The threat of MDR ESKAPE pathogens is particularly high in nosocomial infections, where biofilms formed by bacteria create a protective barrier that makes them highly resistant to antibiotics, complicating the treatment efforts. Scientists are looking at natural and sustainable solutions, as several studies have projected deaths contributed by drug-resistant bacteria to go beyond 50 million by 2050. Many plant-derived metabolites have shown excellent antibacterial and antibiofilm properties that can be tapped for combating superbugs. The present review explores the current status of various studies on antibacterial plant metabolites like alkaloids and flavonoids and their mechanisms in disrupting biofilms and killing bacteria by way of inhibiting key survival strategies of bacteria like motility, quorum-sensing, reactive oxygen species production, and adhesion. These mechanisms were found to be varied in Gram-positive, Gram-negative, and acid-fast bacteria like *Mycobacterium tuberculosis*, which will be discussed in detail. The successful tapping of the benefits of such plant-derived chemicals in combination with evolving techniques of nanotechnology and targeted drug delivery can go a long way in achieving the goal of One Health, which advocates the unity of multiple practices for the optimal health of people, animals, and the environment.

## 1. Introduction

The big conflict between humans and bacterial pathogens has been going on since the introduction of antibiotics in the early twentieth century. Initially, these innovative therapies transformed the medical field by significantly lowering mortality rates related to bacterial infections. Unfortunately, the abuse and misuse of these compounds have resulted in the rise of antibiotic-resistant bacteria, jeopardizing the effectiveness of antibiotic-based treatment strategies. As we approach this critical juncture, the importance of addressing antimicrobial resistance (AMR) becomes pertinent; by 2050, AMR is expected to cause over 10 million deaths per year, surpassing the mortality rates attributed to cancer and other severe diseases [1,2]. Hence, a thorough understanding of the traits and behaviors of resistant bacteria is critical. The rise of multidrug-resistant (MDR) and extensively drug-resistant (XDR) pathogens has crippled the global healthcare sector, multiplying the need for treatment options enormously. Carbapenem-resistant *Pseudomonas aeruginosa* and Carbapenem-resistant *Acinetobacter baumannii* cause significant challenges in treating serious bacterial infections in healthcare settings. Both organisms have developed mechanisms that can resist carbapenem antibiotics; the presence of efflux pumps and biofilm-forming capabilities of *Pseudomonas aeruginosa* complicate the treatment options and raise the risk of death and morbidity in affected patients [3]. Likewise, *Acinetobacter baumannii* resistance is associated with genes associated with β-lactamase synthesis, notably OXA-type enzymes that hydrolyze carbapenems. Both organisms have been found to develop resistance by plasmid-mediated gene transfer, hence improving their survival in antimicrobial-rich environments [4]. Pathogenic bacteria, previously treatable with ordinary antibiotics, have redesigned their defense mechanisms, reducing the efficiency of even last-resort antibiotics such as Methicillin, vancomycin, and carbapenems. Pathogens such as Methicillin-resistant *Staphylococcus aureus* (MRSA) and carbapenem-resistant Enterobacteriaceae (CRE) are notorious in this respect [5,6]. One of the most important mechanisms involved in bacterial resistance is the overexpression of chromosomally encoded efflux pumps, which actively expel a wide range of antibiotics from the cell interior [7]. These pumps not only reduce intracellular antibiotic concentrations but also contribute to a broad-spectrum resistance phenotype, complicating treatment regimens for Gram-negative bacterial infections [8]. The genetic regulation of these efflux systems is frequently linked with other resistance determinants [9].

One of the important blocks for the proper entry of antibiotics into a bacterial cell is the biofilms produced by certain species of bacteria. Biofilm formation activities are intimately related to quorum sensing (QS), a communication mechanism among bacteria that synchronizes group behaviors such as virulence factor expression and matrix creation [10]. As a result, blocking these QS mechanisms has emerged as a prospective target for novel antimicrobial therapies. Natural substances, notably phytochemicals such as alkaloids, flavonoids, terpenoids, and phenolics, have received attention for their significant antibacterial and antibiofilm activities. For example, nanoparticles and plant extracts, such as the leaves of *Lagerstroemia speciosa*, can inhibit biofilm formation by disrupting QS-regulated mechanisms [11,12]. Furthermore, the curative treatment of *Mycobacterium tuberculosis* frequently requires intricate interactions with the host’s immune system. As revealed by Kinsella et al. [13], autophagy plays a vital role in controlling the innate immune response during *M. tuberculosis* infection. Additionally, the effectiveness of therapies may be hampered when dealing with nonreplicating *M. tuberculosis* within the host. Their findings suggest that defects in autophagy may lead to heightened inflammatory responses and hence impair overall infection management. In contrast, other natural products have been shown to diminish QS signals, thus reducing both biofilm maturation and virulence. Nanotechnology breakthroughs have permitted the development of new drug delivery systems targeted at enhancing the stability and bioavailability of phytochemicals at the infection site. Chitosan nanoparticles have emerged as a promising natural antibacterial drug, notably for treating conditions such as omphalitis in chickens caused by a variety of pathogenic bacteria. Chitosan, a biopolymer produced from chitin, has antibacterial capabilities mostly due to its cationic composition, which allows for electrostatic interactions with negatively charged microbial cell surfaces [14]. Nanocarriers, like liposomes and dendrimers, can be designed to protect active plant-derived chemicals from degradation while delivering them into complicated biofilm formations. This method not only improves therapeutic efficacy but also reduces collateral harm to host tissues, emphasizing the need for collaborative efforts to address biofilm-associated infections and MDR pathogens [15]. Finally, the persistence of biofilm-associated illnesses, particularly those caused by ESKAPE bacteria, necessitates a multifaceted approach that blends traditional antibiotics, natural products, and cutting-edge nanotechnology. The current review aims to discuss the threats of AMR, different types of AMR pathogens, and their associated risks and modes of action and then delves into the successful cases of AMR pathogen inhibition by various plant metabolites. Suggestions are also given for the future perspectives on combating the increasing menace of superbugs, with a view to achieving the goal of One Health.

## 2. Threat Posed by Drug-Resistant Bacteria

An example of the Darwinian theories of evolution and of the survival of the fittest can be seen in the case of microorganisms, which have been thriving on this planet in extremely wide-ranging conditions (both biotic and abiotic) since time immemorial. These organisms have always managed to circumvent most types of manmade inhibitions by mutating themselves into becoming more resistant and resilient. Among these, pathogenic microorganisms are of utmost concern, due to their resilience towards various therapeutic interventions. These microorganisms are widely prevalent in the environment and comprise a diverse range of taxonomic categories ranging from unicellular to multicellular eukaryotes, bacteria, and viruses [16]. A few of the popular examples of these pathogenic organisms include *Vibrio cholerae* (which causes cholera and is transmitted through contaminated water or food), *Campylobacter jejuni* (which causes bacterial gastroenteritis and is transmitted through contaminated water, poultry, or milk), *Legionella pneumophila* (which causes Legionnaires’ disease and is transmitted through contaminated aerosols) [16]. Influenza-A viruses (IAVs) have been reported to remain contagious for a long time when present in contaminated sediments and water [17]. Pathogenic microorganisms have thus displayed a long-standing history of being persistent in causing morbidities and mortalities. The average lifespan of humans was thus very limited predominantly due to these reasons, which was significantly improved after the discovery of antibiotics. Penicillin, discovered by Sir Alexander Fleming, still holds potential as a life-saving drug in some instances [18]. Within a few years, this narrative changed drastically, and the successes of antibiotics began to fade due to the emergence of resistant microorganisms. Since then, it has been a constant battle filled with relentless attempts at developing novel and effective strategies against drug-resistant pathogenic microorganisms.

The prevalence of various drug-resistant pathogenic microorganisms in the last few years has distorted the field of health science, impeding various therapeutic developments. AMR in pathogenic microorganisms has emerged as a high-level threat, and these pathogens have also been identified in Arctic regions and on the International Space Station [19]. Additionally, Gram-negative pathogenic microorganisms have been reported to gain resistance towards Colistin, the final armament used in antibiotic-based treatments to safeguard human health against bacterial infections [20]. All these issues have significantly heightened the threats posed by AMR, MDR, XDR and pan-drug-resistant (PDR) Gram-negative pathogens. The World Health Organization (WHO) published the first list of AMR pathogenic microorganisms in 2017, highlighting them as priority pathogens. Further, in 2019, the Centre for Disease Control and Prevention (CDC) had categorized 18 different AMR bacteria and fungi into different levels of public health issues based on their severity, urgency, morbidity and mortality [21]. Expanding this list further, the WHO Bacterial Priority Pathogens List was released in 2024, highlighting 24 pathogens and 15 families of AMR pathogens that include microbes like *Pseudomonas aeruginosa*, *Staphylococcus aureus*, *Neisseria gonorrhoeae* and so on [22]. To date, this concern of AMR is among the top 10 threats to global health [23].

One of the major groups of global high-risk pathogens is the biofilm-forming microbes. These communities of microorganisms are commonly found in nature as being attached to biotic surfaces. They display high levels of resistance towards extreme environmental conditions like salinity, UV radiation, temperature, pressure, antibiotics, etc. Due to this conducive property, they are able to create microbial colonies under varied environmental conditions, displaying high levels of resistance towards different types of factors. Over time, this facilitates the genetic exchange of material between these colonies, facilitating the propagation of their self-protective mechanisms for microbial growth. Various reports suggest that about 80% of these microorganisms have the ability to form biofilms [24]. Problems associated with biofilm-forming pathogens are heavily encountered in the food and health industries, where they serve as persistent sources of contamination and health risk. In the food sector, biofilms also create significant technological challenges leading to the prevention of heat exchange from the surface, an increase in frictional resistance on the surfaces, the mechanical clogging of the food-handling systems and the corrosion of associated metal surfaces [25]. Additionally, biofilms allow the microbes to bind to a wide range of substrates and then develop into mature biofilms within a few days or even hours [26]. A few of these microbes are also capable of forming multi-species biofilms that are exponentially harder to control because of their enhanced stability. Furthermore, in the health sector, biofilms are persistent on the surfaces of medical devices, hospital surfaces and patient tissues, contributing towards persistent infections. A few of these infections, like cystic fibrosis, endocarditis and heart valve infections, even turn out to be lethal [27]. These categories of infections are greatly challenging to treat and cure due to the strong virulence, resistance and tolerance displayed by these microbes even under aggressive treatment regimes.

The threats posed by these microbes are further pertinent when analyzed from the bigger perspective of One Health, where the detrimental effects of AMR pathogenic microorganisms escalate in the ecosystem by affecting plants, animals, humans and the environment. The irresponsible and reckless usage of antibiotics has led to the emergence of AMR microbes severely impacting livestock, human health and agriculture. The overuse of antimicrobials, their improper management, the inadequate control of environmental pollutants and the migration of people and animals infected with resistant pathogens have greatly facilitated the transmittance of AMR genes [28]. For instance, as reported by a recent study in Chile, antimicrobial-resistant *E. coli* were found to be more prevalent in healthy household dogs purchased from pet stores or kennels rather than in those that were adopted [29]. Another similar finding reported the predominant emergence of *Clostridioides difficile* as the causative agent for Inflammatory Bowel Disease (IBD) in animals. These bacteria have now become critical agents in veterinary medicine, particularly affecting wildlife, companion animals and livestock. Research has indicated the transmission of these bacteria to be mediated by factors like antibiotic-mediated dysbiosis, toxin-mediated mechanisms (Tcd-A/B) and environmental spore transmission [30]. These findings emphasize the interconnectedness between animals, humans and the environment, thereby reinforcing the significance of One Health from the perspective of AMR pathogens. A huge amount of research is being directed towards strategizing effective ways to combat AMR microbes that perturb the balance of One Health. Various cutting-edge research in resistome studies have pointed out significant methodologies to control the transmittance of AMR genes. A few of these methodologies include ranking the important AMR hosts and their genes; understanding their transmittance at the interfaces of One Health sectors; identifying the selective pressures contributing towards their emergence, transmittance, and evolution; and understanding the mechanisms that enable organisms to overcome the taxonomic barriers in the transmittance of these genes [31].

The alarming threats of drug resistance posed by pathogenic microbes are further compounded by another class of microorganisms called the ESKAPE pathogens (*Enterococcus faecium*, *S. aureus*, *Klebsiella pneumoniae*, *Acinetobacter baumannii*, *Pseudomonas aeruginosa* and *Enterobacter* sp.). These microorganisms continue to pose major threats to global health due to the wide range of mechanisms they portray, to gain resistance towards various lines of antibacterial treatment regimes and also to disseminate their high-risk clones globally. The WHO first published ESKAPE pathogens as designated priority status pathogens in their list of microbes towards which there is an urgent need for antimicrobial development [32]. As per the WHO updated Bacterial Priority Pathogens List (BPPL) of 2024, the dangerous pathogenic bacteria are grouped under three categories: critical priority, high priority and low priority. The aim of BPPL is to guide the development of the best treatment options and to stop the spread of AMR. Many ESKAPE pathogens have developed resistance mechanisms against a wide range of antibiotics, through ways of genetic mutations and the acquisition of mobile genetic elements [33]. A few of the categories of these antibiotics include lipopeptides, fluoroquinolones, oxazolidinones, macrolides, β-lactam–β-lactamase inhibitor combinations, β-lactams and antibiotics of the last line of defense (like glycopeptides, carbapenems and clinically unfavorable polymyxins) [34].

### 2.1. Characteristic Features of ESKAPE Pathogens

#### 2.1.1. Enterococcus Faecium

This is a very prominent causal organism in various healthcare-associated infections that has also been reported to become increasingly resistant to vancomycin. In the United States, the dissemination of *Enterococcus* first began in the 1980s, which was driven by the third-generation cephalosporins that triggered the emergence of vancomycin and ampicillin-resistant *Enterococcus faecalis*. This was later aggravated through the emergence of vancomycin-resistant *Enterococcus faecium* (VRE*fm*), which also later spread globally [35]. VRE*fm* multilocus sequence types are known to be currently responsible for a significant burden of hospital-acquired infections [36]. When compared to the durations of outbreaks caused by other ESKAPE pathogens, VRE*fm* outbreaks tend to last for longer durations of approximately 11 months [37]. This outbreak is usually catalyzed by antibiotic exposures that enable VRE*fm* to become the predominant species in the gastrointestinal (GI) tract. The treatment significantly relies upon the use of second-line antibiotic regimes (like daptomycin and tigecycline) that are unfortunately associated with high cost and toxicity, along with low efficiency.

#### 2.1.2. Methicillin-Resistant *Staphylococcus aureus* (MRSA)

MRSA was first identified in 1961 as a consequence of the widespread usage of penicillin [38]. MRSA infections generally arise due to either hospital-acquired (HA) or community-acquired (CA) MRSA strains. HA-MRSA strains have generally been associated with bloodstream infections and severe pneumonia, and CA-MRSA strains have generally been associated with soft tissue and skin infections [39]. Another microorganism that was identified in the category of resistant *S. aureus* was BORSA (Borderline Oxacillin-Resistant *S. aureus*), found both in community and hospital settings. BORSA do not characteristically display either Methicillin resistance or sensitivity and are hence extremely difficult to treat due to their non-responsiveness to even high doses of oxacillin [40].

#### 2.1.3. *Klebsiella pneumoniae*

This pathogen, belonging to the order Enterobacterial, has become non-respondent towards the carbapenem and cephalosporin classes of antibiotics, due to the widespread acquisition of drug-resistance genes. High rates of mortality have long been associated with CRE [41], and Carbapenem-resistant *K. pneumoniae* (CRKP) strains are among the most predominant ones among CRE [42]. Recent research also suggests the emergence of AMR hypervirulent *K. pneumoniae* (hvKP) strains worldwide in both high- and low-income regions [43]. One of the characteristic features observed in these strains is the presence of a hypermucoviscous phenotype in the hvKP pathogens [44].

#### 2.1.4. *Acinetobacter baumannii*

This bacterium was historically associated with hot and humid geographic climates [45] but is now typically predominant in hospital environments. High rates of MDR are being reported among *A. baumannii*, especially towards the β-lactam and carbapenem classes of antibiotics. Additionally, with the emergence of PDR microorganisms, the last resort of the polymyxin and carbapenem class of antibiotics is not effective [46].

#### 2.1.5. *Pseudomonas aeruginosa*

This is generally an opportunistic Gram-negative pathogen found associated with severe respiratory infections in patients with diminished immunity. Generally, it is associated with nosocomial infections but is also increasingly known to be associated now with CA infections. *P. aeruginosa* have persistently displayed a broad spectrum of adaptability and plasticity conferred by a repertoire of different regulatory genes that control the pathogen’s ability to chronically persist in the host systems and evade different antibiotics [47].

#### 2.1.6. Enterobacter Species

**Figure 1:**
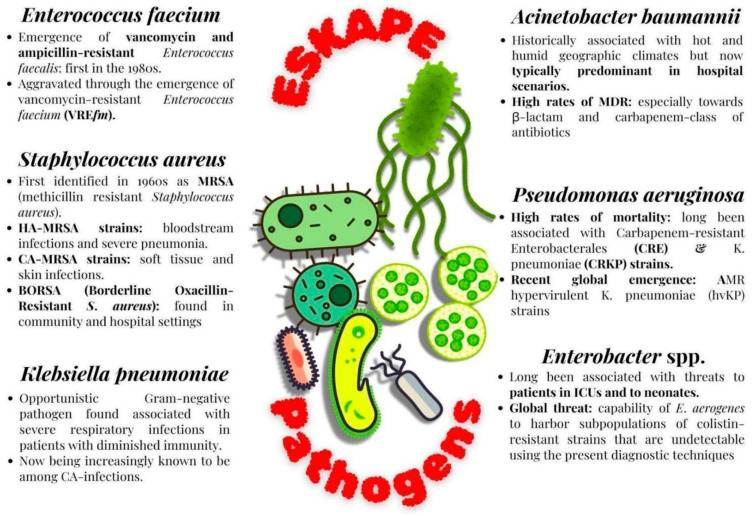
Features of ESKAPE pathogens.

This bacterium has long been associated with significant threats to patients in Intensive Care Units and to neonates [48]. MDR *Enterobacter* sp. hhasbecome an alarming point of concern, especially in the context of HA infections. Furthermore, PDR *E. aerogenes* have also emerged as a serious threat due to their high levels of resistance against Colistin, one of the last-resort antibiotics. This is further complicated by the capability of *E. aerogenes* to harbor subpopulations of colistin-resistant strains that are undetectable using the present diagnostic techniques [49]. A pictorial representation of the significance of ESKAPE pathogens is given below in Figure 1.

## 3. Survival Strategies of Bacteria

Research has extensively probed into understanding the mechanisms of resistance displayed by recalcitrant pathogens, and it has been understood that a few of these predominant mechanisms include the restricted uptake of antibiotics, alterations to the antimicrobial target, efflux, and the degradation of the antimicrobial agent [50]. These resistance mechanisms either are present innately in the microbes or are transferred genetically between and across microbial strains over time. Hence, microorganisms deploy two chief genetic strategies to mitigate antimicrobial attacks, i.e., microbial gene mutations rendering them ineffective towards the antimicrobial compounds or the presence of foreign DNA encoding resistance factors in microbes (catalyzed by conjugation, transduction or transformation) [51]. These processes are further aggravated through horizontal gene transfer (HGT), which contributes towards rapidly disseminating resistance genes in microbes and converting them into AMR strains. Hence, HGT plays a crucial role in enabling pathogen microorganisms to transform into agents of epidemics [52]. Furthermore, three major machineries of resistance, namely tolerance, persistence, and resistance, are detected globally among most resistant pathogenic microbes. Due to these mechanisms, the Minimum Inhibitory Concentration (MIC) of these pathogenic strains has increased considerably [53]. An example of one of the interesting studies corroborating these findings is the one reported in Jiangxi Province. This study, conducted on a pig farm, reported several strains of mobile (plasmid-mediated) colistin-resistance-1 (*mcr*-1)-positive *Escherichia coli* (MCREC) isolated from these animals. These strains were identified to be MDR bearing a gene from the Type-IV Secretion System (T4SS) and antimonite protein, which are known to contribute towards the HGT of virulence factors and antibiotic resistance genes among pathogens [54]. Another interesting study performed in China on the high-risk clone of *ST648* associated with *E. coli* reported the first occurrence of New Delhi Metallo-b-Lactamase (*bla*_NDM-5_) and tigecycline-resistance *tet* (X4) genes, along with several plasmids bearing the capacity of HGT of AMR genes [55].

This persistent problem of fast-growing antibiotic resistance in microorganisms, accompanied by a relatively slow rate of discoveries of novel and effective antibiotic treatment regimes, presents itself as an alarming global threat to humanity. The mechanisms adopted by these bacteria to survive in the presence of antibiotics are a complex array of fascinating and comprehensive phenomena that demonstrate their efficiency in gaining antimicrobial resistance. Various scientific reports have brought to light an interesting niche of antibiotic-resistant microorganisms comprising Bacteria with Reduced or Halted Metabolism (BRHM) [56]. However, due to a dearth in validated methods of identification and quantification of such microbes in clinical samples, there remains a paucity of data corroborating the resistance mechanisms displayed by BRHM. Among other studies on this topic, a few of the prominent ones reported the classification of these resistant microbes based on their survival time in the presence of antibiotics [57]. Based on this categorization, one group of bacteria was those referred to as resistant, which were capable of reproducing in the presence of high concentrations of antibiotics; the second group of bacteria was referred to as tolerant, which had MIC values comparable to that of sensitive bacteria; and the third group of bacteria was referred to as persistent, which had a higher minimum duration of killing values when compared to the tolerant bacteria. However, a few recent findings suggested clinical interpretations of these terms, wherein they report that the terms tolerance and persistence are synonymous [58]. Additionally, they introduced two other terms, triggered persistence and spontaneous persistence [59]. Here, the phenomenon of triggered persistence is signal-induced and comprises a population of tolerant bacteria that may survive when in contact with antibiotics. Another prominent class of resistant microbes is that of the VBNC (Viable but Non-Culturable Bacteria) [60]. These are a subpopulation of bacteria that have the ability to temporarily forfeit their growth on a culture medium on which they previously had the ability to grow. While VBNC cells require a prolonged period and specific conditions of resuscitation after the removal of induced stress, persisters do not display such complex resuscitation dynamics [61]. The theories revolving around the survival strategies of BRHM subpopulation majorly focus on the aspect of bacterial response to negative changes (like the presence of antibiotics, extreme temperatures or pH, etc.) in its extracellular environment. A few of the major mechanisms of bacterial resistance towards antibiotics include a general stress response, an oxidative stress response, a stringent response, ATP depletion and protein aggregation, QS regulation, biofilm formation, the overexpression of efflux pumps, and an SOS response [60].

**Figure 2:**
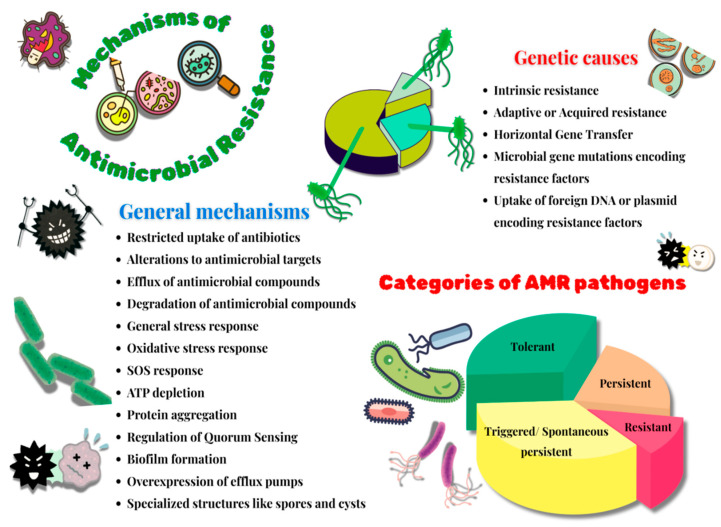
Mechanisms of antimicrobial resistance.

Further theories highlight the evolutionary aspect of bacterial survival strategies. One of the key features of these theories is the trait of intrinsic resistance. This is an evolutionarily conserved trait that enables microbes to survive, grow, and reproduce in the presence of stressors like antibiotics. Its mechanisms are complex and majorly rely on various factors like the species of bacteria, alterations in bacterial membrane permeability, the ability to inactivate β-lactamases or aminoglycoside-modifying enzymes, the presence of efflux pumps. On the other hand, the trait of adaptive or acquired resistance enables the bacteria to survive, grow, and replicate in the presence of specific antibiotics that otherwise suppress the wild-type susceptible strains of the same bacterial species. This trait is gained only by a subset of those bacterial species through genetically induced mechanisms of antibiotic resistance like HGT and mutational events. While the molecular mechanisms of intrinsic and adaptive or acquired resistance are somewhat similar, the crucial difference lies in the fact that the latter is not an evolutionarily conserved phenomenon. Additionally, various environmental cues could lead to the temporary acquisition of antibiotic resistance. The other theories supporting antibacterial resistance demonstrate a variety of mechanisms of survival by BRHM under antibiotic stress, which include A) the presence of specialized genes that induce the transition of bacterial cells into a metabolically suppressed or halted condition and B) bacterial cell structures (like spores and cysts) aiding in conferring antibacterial resistance [62]. A brief overview of these properties is pictorially represented in Figure 2. Microbial survival strategies to gain antimicrobial resistance area topic of immense complexity, intricacy and diversity. Microbes develop novel strategies to evade antimicrobial compounds, and over time, they genetically pass them on to future populations of cells, thereby making this a persistent global problem in the field of health.

## 4. Phytochemicals with Antibacterial Properties

From time immemorial, plant-based therapies have been practiced curing diseases, though scientific validation of the same was not performed. Various plant extracts that have shown potential antibacterial properties have been scientifically proven to possess many bioactive molecules. Research conducted in the past few decades has elucidated their molecular mechanism; some show more inhibition to Gram-positive bacteria and some to Gram-negative bacteria. These differences exist due to inherent structural differences in the cellular architecture of these bacteria.

### 4.1. Phytochemicals Inhibiting Gram-Negative Bacteria

Phytochemicals have sparked widespread interest due to their antibacterial characteristics, notably against Gram-negative bacteria, which are recognized for their resistance to a variety of antimicrobial treatments [63]. This resistance is frequently linked to the complex structure of their cell walls, which feature an outer membrane composed of lipopolysaccharides that acts as a barrier to many antibiotics and phytochemicals [63]. Several studies have shown that particular plant extracts and phytochemical ingredients are effective against Gram-negative bacteria like *E. coli* and *P. aeruginosa*.

The antibacterial activity of five essential oils, viz. eucalyptus, oregano, clove, peppermint and lavender, in combination were studied, which revealed that higher inhibitory effects were achieved in combination than when administered alone, representing their synergistic behavior [64]. Similarly, Gupta et al. [65] investigated the antibacterial activity of different plant extracts and found effective inhibition against a variety of bacterial strains, including Gram-negative kinds. Studies on certain plant extracts have also had notable outcomes. For example, Pangeni et al. [63] found that 31 different plant extracts had positive antibacterial activity against Gram-negative bacteria such as *E. coli* and *P. aeruginosa*.

This study emphasized the role of plant-derived phytochemicals in treating illnesses caused by these resistant bacteria [63]. Similarly, Rauf et al. [66] discovered that extracts from *Diospyros lotus* were effective against Gram-negative bacteria while noting the difficulties connected with producing effective medicines. Significant antibacterial activity was reported for *Canarium patentinervium* preparations against both Gram-positive and Gram-negative bacteria, underlining phytochemicals’ medicinal potential [67]. In conclusion, comprehensive research demonstrates that several phytochemicals have significant antibacterial effects against Gram-negative bacteria. Despite their inherent resistance mechanisms, particular extracts and phytochemical constituents have the ability to operate as effective antibacterial agents, indicating a promising area for future research and application in medicinal therapies.

### 4.2. Phytochemicals Inhibiting Gram-Positive Bacteria

Phytochemicals generated by many medicinal plants have been widely studied for antibacterial properties, particularly against Gram-positive pathogens like *S. aureus* and *E. faecalis*. The success of these natural chemicals is partly due to their distinct chemical structures and modes of action, which allow them to penetrate or damage the bacterial cell wall and interfere with intracellular activities. Terpenoids and fatty acyls have been identified as phytochemicals with strong antibacterial properties. For example, *Pulicaria undulata* extract has been shown to contain terpenoids that displayed high antibacterial activities against *S. aureus*, while substances such as Falcarinol have displayed activity specifically against resistant forms of Gram-positive bacteria [68].

Further investigations have focused on polyphenolic substances, commonly flavonoids, and phenolic acids, which are known for their antioxidant and antibacterial effects. Ethanolic extract of *Linum usitatissimum* has been demonstrated to include alkaloids, flavonoids, and phenolic components that contribute to its antibacterial activity against a variety of Gram-positive pathogens [69]. Furthermore, modified flavonoids such as quercetin and apigenin have been examined for their broad-spectrum antibacterial properties, supporting the notion of structural variations among flavonoids to allow them to target important bacterial processes. *Tanacetum parthenium* extracts have been shown to exhibit high antibacterial action against Gram-positive bacteria such as *S. anginosus* and *S. pyogenes*. The active component parthenolide is thought to target bacterial cells by affecting membrane integrity and interfering with vital metabolic pathways [70]. In a study by Atta et al. [71] the significant synergistic antibacterial activity of plant extracts (*Curcuma amada*, *Terminalia chebula*, and *Nigella sativa*) along with the antibiotic Cefixime against drug-resistant *S. aureus* was explored. The combination treatment notably enhanced bacterial inhibition, underscoring the potential of phytochemicals as powerful adjuvants to antibiotic therapy. Collectively, these findings show the antibacterial activity of phytochemicals being determined by their varied chemical makeup, ranging from terpenoids and essential oils to polyphenols and indoles, which allows for several mechanisms of action. The increased efficacy, along with lesser chances of resistance development when compared to traditional antibiotics, validates the significance of these bioactive chemicals as attractive candidates for future therapeutic applications.

### 4.3. Phytochemicals Inhibiting Mycobacterium Tuberculosis

**Figure 3:**
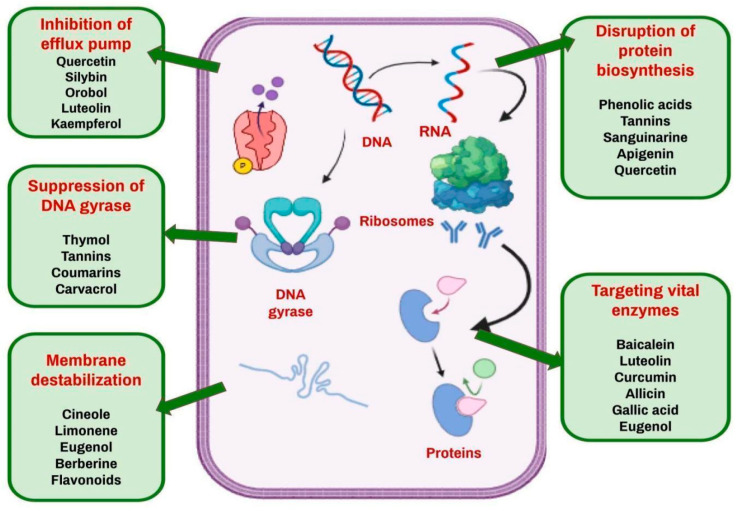
Molecular targets of phytochemicals in bacterial cells.

The application of phytochemicals to defeat *M. tuberculosis* has received a lot of interest, especially considering the urgent need for alternative antitubercular medicines in the face of developing drug resistance and the low number of antitubercular drugs available in the market. Several groups of natural chemicals, including terpenoids, alkaloids, flavonoids, tannins and phenols, have been found to have promising antibacterial activity against *M. tuberculosis* via different modes of action. For example, flavonoids have been found to neutralize reactive free radicals and disrupt nucleic acid synthesis by blocking the topoisomerase enzyme, consequently disrupting the replication machinery of *M. tuberculosis* [72]. *Aspilia pluriceta* extracts have shown good in vitro antitubercular activity [73]. Preliminary investigations on plants like *Nyctanthes arbortristis* indicate that a vast variety of its phytochemicals have the potential to block fatty acid production in this bacterium, which has a rich repertoire of fatty acids, thereby broadening the spectrum of probable molecular targets [74]. Hydroethanolic extracts from traditional Cameroonian medicinal plants, such as *Allium sativum* and *Pentadiplandra brazzeana*, were found to inhibit the growth of multi-resistant *M. tuberculosis* strains [75]. Essential oils have been found to exhibit potent antibacterial activity against tested strains of bacteria; thyme oil has been found to have a very low MIC, less than 9 µL/mL [76]. Various molecular targets of phytochemicals in bacterial cells are depicted in Figure 3.

Studies on the phytochemical profile of *Aloe vera*, which combine traditional knowledge with current analytical chemistry, have demonstrated its promise as an antituberculosis agent, notably against drug-resistant strains. Synthetic alterations of well-known phytochemicals have also been studied for their biological effects. For example, 1,2,4-triazolidine-3-thione derivatives were synthesized and evaluated for antitubercular activity, revealing substantial activity against the *M. tuberculosis* H37Rv strain. Such investigations not only improve our understanding of structure–activity correlations but also help to uncover new therapeutic candidates by using phytochemicals as development scaffolds [77]. This emphasizes the continuous need to research natural chemicals, which may lead to innovative therapeutic approaches against tuberculosis. A few of the mechanisms of action of plant metabolites on specific targets in bacteria are shown in Table 1.

## 5. Phytochemicals with Antibiofilm Properties

The present scenario in the medical domain is such that most of the antibiotics in the market have become inefficient in treating infectious diseases, as the bacterial cells have gained resistance against a broad spectrum of antibiotics. The conditions become worse when these bacterial cells showcase community behavior while forming biofilms. Thus, the approach toward breaking this pattern of emerging resistance is finding its strength in nature’s products [87]. Each plant, in particular, produces trace amounts of secondary metabolites, which are minuscule substances such as alkaloids, phenolics, polyphenols, terpenoids, etc.

### 5.1. Alkaloids

The alkaloids that make up the medicinally active substances fall into a number of classes, including quinolizidines, piperidines, tropanes, purines, pyrrolizidines, imidazoles, isoquinolines, pyrrolidines, and indoles. They are mostly alkaline in nature due to the presence of nitrogen atoms [88]. An alkaloid named reserpine has shown increased antibiofilm activity in *S. aureus* by interacting with the proteins responsible for biofilm formation and virulence, such as icaA, AgrA, Bap, etc. [89]. Of the numerous techniques used by biofilm-forming cells to resist antibiotics, efflux pumps (EP) are found to be crucial. Thus, targeting the EPs using compounds like EP inhibitors (EPI) can be a method that could be studied in great detail to combat resistance. At low concentrations, reserpine prevented the formation of biofilms in clinical isolates of *K. pneumoniae*, indicating that EP inhibition may be the root cause. Caffeoylquinic acid is another example of EPI and has been observed to inactivate biofilm formation in isolates like *S. aureus* and *E. faecalis* [87].

### 5.2. Flavonoids

In a study by Saha et al. [90], sweet orange waste extract was used to study the ability to inhibit biofilm formation in pathogens. The extract was seen to be loaded with flavonoids like hesperidin, narirutin and quercetin. According to the earlier available literature, damage to the cytoplasmic membrane, inhibition of quorum sensing, downregulation of energy metabolism, suppression of nucleic acid synthesis, and obstruction of the active site for bacterial adhesion to surfaces are all factors that contribute towards antibiofilm activity of flavonoids [90]. Faleye et al. [91] suggested that flavonoids have the ability to attenuate adhesion factors in *Vibrio* sp. and also repress multiple virulence properties. Their research also shed light on the fact that the efficacy of antibiofilm components such as 2,2-dihydroxy-4-methoxybenzophenone also depend on the age of the biofilm. A 24 h old biofilm was much easier to target when compared to a 48 h old biofilm. Flavonoids like fisetin and phloretin were shown to target biofilms formed by MDR strains of *A. baumannii* at just 50 μg/mL concentration [92].

### 5.3. Saponins

By downregulating the transcription of the biofilm-associated genes, such as *srtA*, *fbsC*, *neuA*, and *cpsE*, tea saponins were seen to prevent the formation of biofilms in *Streptococcus agalactiae* (GBS), a highly infectious bacterium that can cause meningitis in newborns, as well as pneumonia and septicemia [93]. Recent research by Li et al. [94] suggests that 0.16 mg/mL of saponins could downregulate *ALS3* and *ECE1* genes, which results in a dramatic decrease in hydrophobicity and adhesiveness of the surface, which would be disadvantageous for biofilm formation [94]. In a study by Fink and Filip, quillaja saponin, which is a natural saponin, was compared with sodium dodecyl sulphate, a synthetic detergent, for various properties. In the study, quillaja saponin showed better antibiofilm activity and less toxicity than its synthetic counterpart, making it a viable replacement [95]. In the realm of drug delivery systems and the rise in the emergence of multiple drug-resistant species, silver nanoparticles (AgNPs) have a pivotal role to play. Many scientists today are using the green synthesis method to produce nanoparticles. Saponins were used to synthesize AgNPs in a study by Adnan et al., and it was observed that by disrupting QS-signaling molecules, which are essential for bacterial virulence and pathogenicity, the saponin-derived AgNPs were successfully able to inhibit biofilm formation [96].

### 5.4. Tannins

It has been reported that certain tannins have a mode of action specifically targeting biofilms. For example, tannic acid has been shown to suppress QS in *P. aeruginosa* and to induce transglycosylase activity in *S. aureus*. More significantly, the antibiofilm action against Gram-negative bacteria can be enhanced by altering the tannins with hydroxy-N,N,N-trimethylpropanyl-3-aminium chloride (C_3_NMe_3_Cl-0.5) and PEG500-0.05 [97]. In another study conducted by Qin et al. [98], by lowering EPS and eDNA secretion, tannins isolated from *Penthorum chinense* Pursh prevented the formation of biofilms.

### 5.5. Phenolics

The Cl or OH groups in phenolic molecules appear to improve both the rupture of preformed biofilms and the suppression of biofilm development [99]. Protocatechuic acid and p-hydroxybenzoic acid present in the ethanolic extract of *Inonotus obliquus* were shown to contribute towards antibiofilm activity against *P. aeruginosa* by altering the bacterial flagella and pili surface attachment and their swimming and twitching capacity. In addition to influencing motility, the extract has also been noted for its anti-quorum sensing activities [100]. Numerous polyphenols based on catechins block bacterial glycosyltransferase, which is one of the essential virulence factors and plays a part in the manufacture of glucan polysaccharide, a key component of the biofilm matrix, thus preventing oral biofilms in the form of dental plaques [101].

Phytochemicals demonstrate strong antibacterial properties, weaken the structural integrity of biofilms, and disturb bacterial communication pathways. Additionally, their use alongside traditional antibiotics has been shown to improve treatment results. Thus, phytochemicals could be valuable in anti-biofilm strategies, potentially complementing or even replacing conventional treatments, provided they are used at the right therapeutic doses and through effective delivery methods. This emphasizes the importance of further research into their clinical potential for treating infections complicated by biofilms [102]. Some of the mechanisms of action of metabolites possessing antibiofilm activity are depicted in Table 2.

A study by Albano et al. [115] showcased a novel strategy to combat drug-resistant *Mycobacterium abscessus* by using polymer-stabilized phytochemical nanoemulsions. The nanoemulsion consisted of phytochemicals such as carvacrol and eugenol. The cationic polymer system was found to engage with the negatively charged components of the bacterial surface, promoting deeper penetration into biofilms and thereby targeting the pathogen. This approach showed promising results in both in vitro biofilm models and in vivo wound infection models.

## 6. Mechanisms Disrupting Bacterial Growth and Biofilm Formation

With the discovery of penicillin, many scientists shifted their focus to isolating natural compounds from microorganisms to target various bacteria. Many actinomycetes were in the limelight during the 1940s, and finally, by 1944, streptomycin was isolated from *Streptomyces griseus* and was used against various pathogens. However, scientists did not have to wait too long to observe a resistance pattern in pathogens against these antibiotics [116]. The antibiotics that were discovered till then targeted the synthesis of cell walls, proteins, nucleic acids, or folic acid in pathogens. All of these are crucial for the survival of the organisms. Therefore, it is not surprising that the pathogens tried to evade these mechanisms by numerous strategies. Most of the resistance pattern was observed due to the ability of the pathogens to produce an enzyme called β-lactamase, which would degrade the β-lactam ring in antibiotics like penicillin and cephalosporin. The genes responsible for the production of β-lactamase enzymes are most often plasmid-encoded and are transferred to other bacteria easily when they are biofilm formers [117].

Bacterial cells, when in a biofilm, accumulate in a matrix of extracellular polymeric substances (EPSs). By acting as a physical barrier, EPSs keep bacteria and antimicrobial agents from interacting. Antibiotics are considered inactive, and their ability to invade the matrix is hindered by the integrated enzymes in the matrix and charged polymeric components. The communication between bacteria in a biofilm is well synchronized through a phenomenon called quorum sensing. It is an ability that some bacterial species possess to sense the population density in their immediate environment. This sensing is made possible by autoinducer molecules such as acyl homoserine lactones in Gram-negative bacteria and small peptides in Gram-positive bacteria that are secreted by the cells into their surroundings. As the concentration of the autoinducers in the environment increases, these molecules return into the cells to activate certain proteins that allow the expression of genes coding for some factors like bioluminescence, virulence, biofilm formation, etc. [118]. To avoid the end result of quorum sensing, a technique called Quorum Quenching (QQ) is currently employed. Enzymes can disrupt AHL-mediated QS by degrading AHLs. Depending on how they function, three different types of enzymes can make AHLs inactive: (i) lactonases open the lactone ring; (ii) oxidoreductases either oxidize the acyl chain of AHLs or change 3-oxo-AHLs into their corresponding 3-hydroxy-AHL counterparts; (iii) acylases, also called amidases, hydrolyze the amide bond of AHLs and transform it into the corresponding fatty acid and homoserine lactone [119]. To date, the majority of Quorum-Quenching Enzymes (QQEs) have been identified as lactonases [120]. A QQE called AiiA and a QSI called G1 were combined in a study by Fong et al. [121]. In the *P. aeruginosa* PAO1 strain, which possesses both *las* and *rhl* circuits, G1 was found to repress the las system but not the rhl system.

**Figure 4:**
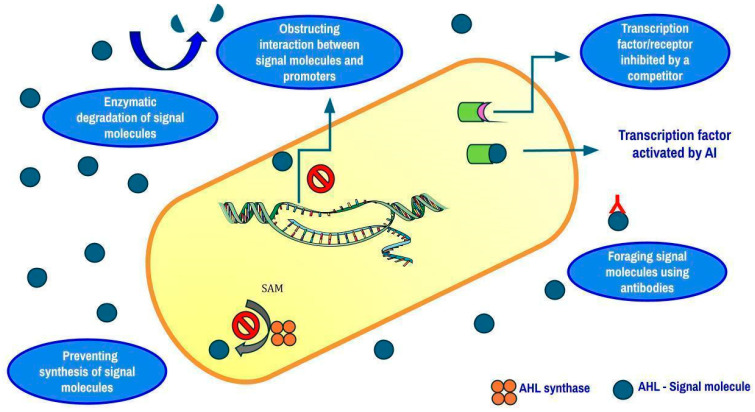
Mechanisms of biofilm disruption in Gram-negative bacteria.

However, when lasR was altered, G1 was able to effectively inhibit the *rhl* system. The enzyme produced by the *aiiA* gene, which was discovered in Gram-positive Bacillus species, can deactivate AHL signals and suppress QS signaling by hydrolyzing the ester link of the homoserine lactone ring. It was suggested that AHL lactonase (AiiA) inhibits the generation of virulence factors and QS signals, enabling the host defense mechanisms to stop and eradicate the bacterial infection [121]. In order to ascertain how certain Egyptian medicinal plants suppressed *P. aeruginosa*’s QS signaling pathway, Naga et al. conducted a study. *Mangifera indica* had the most quorum-sensing inhibitory action against *C. violaceum* ATCC 12472 among the plants that were tested. After the extraction and identification of four pure chemicals, methyl gallate (MG) showed the strongest QSI [122]. It has been demonstrated that a number of QSIs function in different ways (Figure 4), such as (i) blocking the synthesis of signal molecules, (ii) breaking down signal molecules such as lactonase enzymatically, (iii) making it difficult for signaling molecules to bind to receptors, (iv) blocking the interaction between signal molecules and gene promoters to prevent the expression of genes, and (v) foraging AIs by using macromolecules and antibodies [123]. To assess the efficacy of phytochemicals against biofilm-forming pathogens, three modes of measurement are taken into consideration, viz., MIC, MBIC (Minimum Biofilm Inhibitory Concentration), and MBEC (Minimum Biofilm Eradication Concentration). MBIC is effective mostly against bacterial pathogens that are in the initial stages of biofilm formation. On the contrary, MBEC targets mature biofilms [124]. A list of a few natural compounds and their respective MIC, MBIC, and MBEC values against biofilm-forming pathogens are depicted in Table 3.

Despite all the advantages of phytochemicals, occasionally, they can show cytotoxic effects [128]. In a study by Anywar et al. [129], two plants, *Warburgia ugandensis* and *Albizia coriaria*, showed high cytotoxicity levels and adverse effects on individuals who had failed to follow the usage instructions. Hence, the proper usage of herbal medicines is crucial to avoid potential toxic side effects [129]. Since phytochemicals are compounds that target bacterial pathogens like antibiotics, eventually, they could resist these potent phytochemicals. And in such situations, new formulations of phytochemicals can help in targeting the pathogens [115].

There were few in vivo studies on ways to assess biofilm formation and inhibition to control zoonotic diseases. The growing antibiotic resistance in a biofilm-forming pathogen *Pasteurella multocida*, which is a key secondary pathogen in bovine respiratory diseases, was explored in great detail. The plant-derived compounds thymol and berberine demonstrated strong inhibitory effects on biofilm formation and significantly suppressed the expression of associated genes, presenting them as promising candidates for the treatment of *P. multocida* infections linked to biofilms [130]. The ability of *Streptococcus dysgalactiae* sub sp. *dysgalactiae* to form biofilms in vivo in murine models and ex vivo on human-skin-derived cells was assessed in another study. This pathogen is known for causing bovine mastitis, which affects the dairy industry negatively. It was proven that *S. dysgalactiae* was able to establish a robust biofilm on catheter surfaces implanted in mouse models and that genes governing virulence were upregulated in biofilms formed in living hosts when compared to biofilms in laboratory conditions. This finding emphasizes its potential as a zoonotic agent capable of contributing to human diseases, including device-associated infections and cellulitis [131].

## 7. Limitations of Current Antibacterial Strategies

The discovery of antibiotics created a paradigm shift in the era of health science, bringing a ray of hope in the fight against deadly pathogenic microbes. There was a steady decline in the morbidity and mortality caused by pathogenic bacteria, due to a number of antibiotics with varied target hits on bacteria in clinical use. Over the years, however, the overuse and misuse of antibiotics have led to the emergence of AMR superbugs like MDR, PDR, ESKAPE, and XDR pathogens that are steadfast and hard to tackle using present-day commercial antibiotics. Another problem with AMR is the rate of horizontal gene transfer that these pathogens perform. Antibiotic resistance genes are transferred to different species, making them also resistant to antibiotics. With the widespread use of antibiotics, mostly as prophylactic measures, especially in animal farms, there exists a perfect setting for these superbugs to spread across several species barriers, making the situation even more worrisome. When it comes to biofilm-forming bacteria, the challenges are multipronged—the physical barrier of EPS in biofilms, their ability to neutralize the antibiotics and the low metabolic rate of such bacteria thereby give less chance for the antibiotic to act on dividing cells. When analyzing the fast pace at which AMR pathogens are evolving, one can easily understand that there exists a discord between the pace of evolution of superbugs and the rates at which novel chemically synthesized and purified antibiotics are developed to combat these AMR pathogens. Phytocompounds are untapped reservoirs of potential biomolecules with therapeutic properties. There is a lacuna in research that can help salvage the beneficial properties of phytocompounds to treat drug-resistant pathogenic microorganisms. Recent research has compared the efficacy of extracts and nanoparticles of *Psidium guajava* and *Syzygium jambos* in antibiofilm activity and has also synthesized a gel formulation that depicts good wound-healing activity too [132]. Thus, by synchronizing the sources of traditional knowledge systems of using plants for their biological properties, with new-age techniques like nanotechnology and targeted drug delivery systems, we could develop robust therapeutic strategies to overcome the rapidly evolving problems associated with AMR pathogens and other superbugs.

## 8. Conclusions and Future Prospects

The dangers posed in the healthcare sector by dangerous, multidrug-resistant and biofilm forming bacterial pathogens are indeed a global challenge. Scientists, doctors and healthcare workers have to be prepared for emergencies like serious outbreaks of new strains of pathogens resistant to many of the prevailing drugs. It is always a good idea to search in nature for solutions to such problems. Many phytochemicals that are secondary metabolites in plants, produced mainly in response to stress, have been proven to inhibit biofilm formation and even dismantle mature biofilms, making bacterial cells vulnerable and more susceptible to treatment. This ability to disrupt biofilm development is of particular value in the treatment of chronic infections and nosocomial infections, like those associated with medical devices, implants, ICU instruments, etc. There is a dire need to explore novel and robust treatment strategies to win the war against biofilm-forming bacteria by a three-pronged approach of the effective delivery of antibacterial agents, breaking the physical barriers of the biofilm matrix and tackling the menace of bacterial resistance.

The diverse mechanisms of action of the phytochemicals are really advantageous, as they lessen the likelihood of the development of resistance by the pathogens. Many such compounds are ingredients in traditional knowledge systems, thereby providing a wealth of knowledge and accessibility for research, their only drawback being a lack of scientific validation in the case of many bioactive molecules. Ongoing research on the synergistic effects of phytochemicals with conventional antibiotics could lead to potential combination therapies for combating multidrug-resistant bacteria. Targeting crucial biofilm formation pathways like quorum sensing and enhancing antibiotic delivery using new carriers are potential solutions to address this global health dilemma posed by drug-resistant bacteria. Nanotechnology also offers tremendous possibilities, in both the synthesis and the delivery of such plant chemical molecules, to combat deadly bacteria like the ESKAPE pathogens. Many sustainable green synthesis methods have also been formulated for nanoparticles, which can be a component of the armor against biofilm-forming bacteria. However, scaling up this technique at an industrial level presents several challenges, viz., variations in the phytochemical composition in plants, the complexity in standardizing the extraction procedures, and difficulties in maintaining consistency of the nanoparticle size and shape during large-scale production. Therefore, optimizing the conditions used for synthesis is essential to ensure quality control. Another challenge would be the supply chain issues that could be caused by seasonal variations and limited availability of plant materials. However, phytochemicals with their antibacterial and antibiofilm properties, coupled with their natural origins, represent a valuable tool in the fight against MDR bacteria and could prove to be a more sustainable option for achieving the goal of One Health in an era where antibiotic resistance looms large. Their practical application is most often hindered due to their limitations in bioavailability, metabolic stability, and in vivo efficacy, thereby demanding further research into delivery systems and formulation strategies.

## Figures and Tables

**Table 1:** Mechanisms of action of plant metabolites on specific targets in bacteria.

Phytochemicals	Target Bacteria	Mode of Action	References
Luteolin (bioflavonoid)	*Staphylococcus aureus*	Kills both planktonic and biofilm-forming bacteria by disrupting the cell membrane and inhibiting biofilm formation	[78]
Catechols (7,8-dihydroxyflavone, myricetin, and luteolin)	*Klebsiella pneumoniae*	Disrupt iron homeostasis in bacteria—ferric ions were reduced to ferrous form—and increases ROS production, leading to cell ferroptosis	[79]
Flavonoids, phenols, saponins, steroids, terpenoids	*Pseudomonas aeruginosa* strain NAPCC-1 and strain KAR12.	Cause membrane destabilization and interrupts the life cycle by targeting important enzymes.	[80]
Alkaloids, quinones, steroids, terpenes, saponins, flavonoids, tannins	20 bacterial strains representing 13 species	Disrupt bacterial membranes, leading to leakage of cellular contents and inhibition of essential cellular functions.	[81]
Linoleic acid, Lupeol, Epi-psi-Taraxastanonol	*Staphylococcus aureus*, *Pseudomonas aeruginosa*	Disturb bacterial cell membrane integrity, causing the leakage of cellular contents.	[82]
Eugenol, essential oils	*Staphylococcus aureus* (multi-resistant strain) *Escherichia coli* (multi-resistant strain)	Inhibit protein biosynthesis and disturbs the bacterial cell membrane.	[83]
Alkaloids, flavonoids, phenols, tannins, terpenoids	*Enterobacter faecalis,* *Escherichia coli*, *Pseudomonas aeruginosa*, *Klebsiella pneumoniae*	Breach the outer membrane to interfere with essential intracellular processes.	[84]
Stigmasterol, β-amyrin, 3-O-β-D-glucopyranosylstigmasterol, 3-O-β-galactopyranosyl-(1→4)-β-D-galactopyranosyl-oleanolic acid	*Klebsiella pneumoniae*, *Pseudomonas aeruginosa* and *Escherichia coli*	Inhibit bacterial efflux pumps, increasing intracellular antibiotic levels and combating multidrug resistance	[85]
Alcarinol, spathulenol, and phytol	Vancomycin-resistant *Enterococci*, *M. tuberculosis* Methicillin-resistant *Staphylococcus aureus*	Disrupt bacterial membranes and inhibit enzymes, causing cell death	[86]

**Table 2:** Mode of action of plant metabolites with antibiofilm activity.

Phytochemicals	Compounds	Mode of Action	Reference
Alkaloids	Berberine	Decreases the transcription of AgrA and thereby targets biofilm dispersal in *S. aureus*	[103]
	Sanguinarine	Inhibits the biofilm formation in carbapenem-resistant *Serratia marcescens* (CRSM) by disrupting the cell-membrane integrity.	[104]
	Piperine	Downregulates *icaA* gene, resulting in a reduction in extracellular polysaccharide production, a reduction in cell-surface hydrophobicity, and the regulation of AgrA protein, resulting in interference with quorum-sensing and microbial motility components in Methicillin-resistant *Staphylococcus aureus* (MRSA)	[105]
Flavonoids	Quercetin Myricetin Scutellarein	Inhibits the assembly of Bap-related amyloid-like structures in *S. aureus* and thus prevents biofilm formation	[106]
	Epigallocatechin gallate	Prevents the formation of extracellular polysaccharide	[107]
Saponins	β-Aescin	Alters the hydrophobicity of the cell surface and thereby eradicates biofilms	[108]
	*Quillaja saponaria* saponins	Acts as a natural detergent to eradicate biofilms and thus is used in wastewater treatment	[109]
Tannins	Ellagic acid	Disrupts bacterial redox balance via WrbA, thereby impairing biofilm formation.	[110]
	Proanthocyanidin	Chelates iron, thereby preventing the biofilm from maturing completely or allowing only a thin layer of biofilm to form that is not very resistant to antibiotics	[111]
Phenolics	Curcumin	Inhibits pellicle and pili formation and restricts motility	[102]
	Resveratrol	Inhibits motility and quorum sensing in *Aeromonas hydrophila*	[112]
Terpenes	Carvacrol	Inhibits quorum sensing in *E. coli*	[113]
	Thymol	Inhibits quorum sensing and production of PIA	[114]

**Table 3:** Minimum Inhibitory Concentration (MIC), Minimum Biofilm Inhibitory Concentration (MBIC) and Minimum Biofilm Eradication Concentration (MBEC) (mg/mL) of phytochemicals against pathogenic bacteria.

Pathogens	Natural Compounds	MIC	MBIC	MBEC	Reference
*E. coli*	Thymol	2.4 mg/mL	0.3 mg/mL	1.2 mg/mL	[124]
	Carvacrol	0.6 mg/mL	0.6 mg/mL	2.4 mg/mL	
	p-Cymene	0.7 mg/mL	0.1 mg/mL	0.5 mg/mL	
	γ-Terpinine	2.2 mg/mL	0.5 mg/mL	2.1 mg/mL	
*P. aeruginosa*	Curcumin	417.68 μg/mL	565.89 μg/mL	>2048 μg/mL	[125]
	Piperine	>1024 μg/mL	>2048 μg/mL	>2048 μg/mL	
	Quercetin	77.47 μg/mL	52.21 μg/mL	195.37 μg/mL	
	Plumbagin	644.92 μg/mL	1024–2048 μg/mL	>2048 μg/mL	
*A. baumannii*	Methanol Extract from *Acanthus polystachyus* Delile	0.5 mg/mL	0.5 mg/mL	-	[126]
	Essential Oil from *Acanthus polystachyus* Delile	0.31 mg/mL	0.31 mg/mL	-	
Methicillin-Susceptible *Staphylococcus aureus* (MSSA) and Methicillin-Resistant *Staphylococcus aureus* (MRSA) strains	Volatile phytochemicals Carvacrol (CAR) and Thymol (THY)	CAR: 128–203.2 μg/mL for MSSA and 362–1024 μg/mL for MRSA THY: 256–724.01 μg/mL for MSSA and 512.0 ≥ 2048 μg/mL for MRSA	-	-	[127]
*K. pneumoniae*	*Thyme* essential oils	6 μL/mL	-	-	[76]
	*Lavender* essential oils	6 μL/mL	-	-	
	*Mint* essential oils	6 μL/mL	-	-	

## Data Availability

No new data were created or analyzed in this study.

## References

[1] Zhou S., Barbosa C., Woods R. (2020). Why is Preventing Antibiotic Resistance So Hard? Analysis of Failed Resistance Management. Evol. Med. Public Health.

[2] Zeiler M., Melander R., Melander C. (2020). Second-Generation Meridianin Analogues Inhibit the Formation of *Mycobacterium smegmatis* Biofilms and Sensitize Polymyxin-Resistant Gram-Negative Bacteria to Colistin. ChemMedChem.

[3] Wang Z., Tian W., Sun S., Chen X., Wang H. (2023). Genomic and Proteomic Analysis of Pseudomonas Aeruginosa Isolated from Industrial Wastewater to Assess its Resistance to Antibiotics. Separations.

[4] Li J., Li Y., Cao X., Zheng J., Zhang Y., Xie H., Li C., Liu C., Shen H. (2023). Genome-Wide Identification and Oxacillinase OXA Distribution Characteristics of *Acinetobacter* spp. Based on a Global Database. Front. Microbiol..

[5] Alsubaie M., Alsuheili A., Aljehani M., Alothman A., Alzahrani A., Mohammedfadel H., Alnajjar A. (2023). Antibiotic Resistance Patterns of Pediatric Community-Acquired Urinary Tract Infections in a Tertiary Care Center in Jeddah, Saudi Arabia. J. Infect. Dev. Ctries..

[6] Zhao A., Sun J., Liu Y. (2023). Understanding Bacterial Biofilms: From Definition to Treatment Strategies. Front. Cell. Infect. Microbiol..

[7] Mukhopadhyay S., Singh M., Ghosh M.M., Chakrabarti S., Ganguli S. (2024). Comparative Genomics and Characterization of *Shigella flexneri* Isolated from Urban Wastewater. Microbes Environ..

[8] Wójcicki M., Świder O., Daniluk K.J., Średnicka P., Kowalczyk M., Roszko M., Juszczuk-Kubiak E. (2021). Transcriptional Regulation of the Multiple Resistance Mechanisms in *Salmonella*—A Review. Pathogens.

[9] Li W., Hu J., Li L., Zhang M., Cui Q., Ma Y., Wang M. (2022). New Mutations in *cls* Lead to Daptomycin Resistance in a Clinical Vancomycin- and Daptomycin-Resistant *Enterococcus faecium* Strain. Front. Microbiol..

[10] Banerjee D.K., Biswas P., Mazumder K., Palai S., Hossain C.M., Karmakar S., Biswas K. (2024). Exploration of Phytochemicals as Anti-Biofilm Agents Against Pathogenic Bacteria: Their Potential and Challenges. Infect. Disord. Drug Targets.

[11] Kanwar K., Thakur P., Azmi W. (2018). Use of Phytochemicals as Emerging Strategy for Control of Biofilm Formed by Pathogens. Ann. Phytomed..

[12] Hannan A., Du X.X., Maqbool B., Khan A. (2024). Nanoparticles as potent allies in combating antibiotic resistance: A promising frontier in antimicrobial therapy. Pak. Vet. J..

[13] Kinsella R.L., Kimmey J.M., Smirnov A., Woodson R., Gaggioli M.R., Chavez S.M., Kreamalmeyer D., Stallings C.L. (2023). Autophagy Prevents Early Proinflammatory Responses and Neutrophil Recruitment during *Mycobacterium tuberculosis* Infection Without Affecting Pathogen Burden in Macrophages. PLoS Biol..

[14] Saad N., El-Abasy M.A., El-Khayat F., Ali N.G., Ismail M.M. (2023). Efficacy of Chitosan Nanoparticles as a Natural Antibacterial Agent Against Pathogenic Bacteria Causing Omphalitis in Poultry. Pak. Vet. J..

[15] Rehman Z.U., Leiknes T. (2018). Quorum-Quenching Bacteria Isolated from Red Sea Sediments Reduce Biofilm Formation by *Pseudomonas aeruginosa*. Front. Microbiol..

[16] Balloux F., van Dorp L. (2017). Q&A: What are Pathogens, and What Have They Done to and for us?. BMC Biol..

[17] Reeves A.B., Ramey A.M., Koch J.C., Poulson R.L., Stallknecht D.E. (2020). Field-Based Method for Assessing Duration of Infectivity for Influenza A Viruses in the Environment. J. Virol. Methods.

[18] Spellberg B., Bartlett J., Wunderink R., Gilbert D.N. (2015). Novel Approaches Are Needed to Develop Tomorrow’s Antibacterial Therapies. Am. J. Respir. Crit. Care Med..

[19] McCann C.M., Christgen B., Roberts J.A., Su J.Q., Arnold K.E., Gray N.D., Graham D.W. (2019). Understanding Drivers of Antibiotic Resistance Genes in High Arctic Soil Ecosystems. Environ. Int..

[20] Aghapour Z., Gholizadeh P., Ganbarov K., Bialvaei A.Z., Mahmood S.S., Tanomand A., Kafil H.S. (2019). Molecular Mechanisms Related to Colistin Resistance in *Enterobacteriaceae*. Infect. Drug Resist..

[21] Centers for Disease Control and Prevention (CDC), National Center for Emerging and Zoonotic Infectious Diseases (NCEZID), Division of Healthcare Quality Promotion (DHQP) (2020). 2019 AR Threats Report.

[22] World Health Organization (2024). WHO Bacterial Priority Pathogens List, 2024: Bacterial Pathogens of Public Health Importance, to Guide Research, Development, and Strategies to Prevent and Control Antimicrobial Resistance.

[23] World Health Organization (2022). Global Antimicrobial Resistance and Use Surveillance System (GLASS) Report 2022.

[24] Flemming H.C., Wuertz S. (2019). Bacteria and Archaea on Earth and Their Abundance in Biofilms. Nat. Rev. Microbiol..

[25] Meesilp N., Mesil N. (2019). Effect of Microbial Sanitizers for Reducing Biofilm Formation of *Staphylococcus aureus* and *Pseudomonas aeruginosa* on Stainless Steel by Cultivation with UHT Milk. Food Sci. Biotechnol..

[26] Carrascosa C., Raheem D., Ramos F., Saraiva A., Raposo A. (2021). Microbial Biofilms in the Food Industry—A Comprehensive Review. Int. J. Environ. Res. Public Health.

[27] Sharahi J.Y., Azimi T., Shariati A., Safari H., Tehrani M.K., Hashemi A. (2019). Advanced Strategies for Combating Bacterial Biofilms. J. Cell. Physiol..

[28] Velazquez-Meza M.E., Galarde-López M., Carrillo-Quiróz B., Alpuche-Aranda C.M. (2022). Antimicrobial resistance: One health approach. Vet. World.

[29] Zelaya C.A., Arriagada G., Medina R., Escobar B., Sánchez F., Galarce N., Lapierre L. (2025). The Risk Factors Associated with the Carriage to Critical Antimicrobial-Resistant *Escherichia coli* in Healthy Household Dogs: A One Health Perspective. Animals.

[30] Salvarani F.M., Oliveira H.G.d.S., Uzal F.A. (2025). *Clostridioides difficile* in Animal Inflammatory Bowel Disease: A One Health Perspective on Emerging Zoonotic Threats. Microorganisms.

[31] Kim D.W., Cha C.J. (2021). Antibiotic resistome from the One-Health perspective: Understanding and controlling antimicrobial resistance transmission. Exp. Mol. Med..

[32] Tacconelli E. (2017). Global Priority List of Antibiotic-Resistant Bacteria to Guide Research, Discovery, and Development.

[33] Beatson S.A., Walker M.J. (2014). Tracking Antibiotic Resistance. Science.

[34] Naylor N.R., Atun R., Zhu N., Kulasabanathan K., Silva S., Chatterjee A., Robotham J.V. (2018). Estimating the Burden of Antimicrobial Resistance: A Systematic Literature Review. Antimicrob. Resist. Infect. Control.

[35] Murray B.E. (1990). The Life and Times of the *Enterococcus*. Clin. Microbiol. Rev..

[36] Lee T., Pang S., Abraham S., Coombs G.W. (2019). Antimicrobial-Resistant CC17 *Enterococcus faecium*: The Past, the Present and the Future. J. Glob. Antimicrob. Resist..

[37] Satilmis L., Vanhems P., Benet T. (2016). Outbreaks of Vancomycin-Resistant *Enterococci* in Hospital Settings: A Systematic Review and Calculation of the Basic Reproductive Number. Infect. Control Hosp. Epidemiol..

[38] Eriksen K.R. (1961). “Celbenin”-Resistant Staphylococci. Ugeskr. Laeger.

[39] DeLeo F.R., Otto M., Kreiswirth B.N., Chambers H.F. (2010). Community-Associated Meticillin-Resistant *Staphylococcus aureus*. Lancet.

[40] Skinner S., Murray M., Walus T., Karlowsky J.A. (2009). Failure of Cloxacillin in Treatment of a Patient with Borderline Oxacillin-Resistant *Staphylococcus aureus* Endocarditis. J. Clin. Microbiol..

[41] Tzouvelekis L.S., Markogiannakis A., Psichogiou M., Tassios P.T., Daikos G.L. (2012). Carbapenemases in *Klebsiella pneumoniae* and Other *Enterobacteriaceae*: An Evolving Crisis of Global Dimensions. Clin. Microbiol. Rev..

[42] U.S. Department of Health and Human Services, Centers for Disease Control and Prevention (2013). Antibiotic Resistance Threats in the United States.

[43] Krapp F., Morris A.R., Ozer E.A., Hauser A.R. (2017). Virulence Characteristics of Carbapenem-Resistant *Klebsiella pneumoniae* Strains from Patients with Necrotizing Skin and Soft Tissue Infections. Sci. Rep..

[44] Li W., Sun G., Yu Y., Li N., Chen M., Jin R., Wu H. (2014). Increasing Occurrence of Antimicrobial-Resistant Hypervirulent (Hypermucoviscous) *Klebsiella pneumoniae* Isolates in China. Clin. Infect. Dis..

[45] Ibrahim M.E. (2019). Prevalence of *Acinetobacter baumannii* in Saudi Arabia: Risk Factors, Antimicrobial Resistance Patterns and Mechanisms of Carbapenem Resistance. Ann. Clin. Microbiol. Antimicrob..

[46] Xie R., Zhang X.D., Zhao Q., Peng B., Zheng J. (2018). Analysis of Global Prevalence of Antibiotic Resistance in *Acinetobacter baumannii* Infections Disclosed a Faster Increase in OECD Countries. Emerg. Microbes Infect..

[47] Gellatly S.L., Hancock R.E.W. (2013). *Pseudomonas aeruginosa*: New Insights into Pathogenesis and Host Defenses. Pathog. Dis..

[48] Davin-Regli A., Pagès J.M. (2015). *Enterobacter aerogenes* and *Enterobacter cloacae*; Versatile Bacterial Pathogens Confronting Antibiotic Treatment. Front. Microbiol..

[49] Band V.I., Crispell E.K., Napier B.A., Herrera C.M., Tharp G.K., Vavikolanu K., Weiss D.S. (2016). Antibiotic Failure Mediated by a Resistant Subpopulation in *Enterobacter cloacae*. Nat. Microbiol..

[50] Baker S.J., Payne D.J., Rappuoli R., De Gregorio E. (2018). Technologies to Address Antimicrobial Resistance. Proc. Natl. Acad. Sci. USA.

[51] Munita J.M., Arias C.A. (2016). Mechanisms of Antibiotic Resistance. Virulence Mechanisms of Bacterial Pathogens.

[52] Lerminiaux N.A., Cameron A.D.S. (2019). Horizontal Transfer of Antibiotic Resistance Genes in Clinical Environments. Can. J. Microbiol..

[53] Pacios O., Blasco L., Bleriot I., Fernandez-Garcia L., González Bardanca M., Ambroa A., Tomás M. (2020). Strategies to Combat Multidrug-Resistant and Persistent Infectious Diseases. Antibiotics.

[54] Wu Y., Wang C.H., Li X., Li F., Jiang M.L., Liu Z.K., Li J.Y. (2024). Characteristics of The Plasmid-Mediated Colistin-Resistance Gene Mcr-1 In *Escherichia Coli* Isolated from Pig Farm in Jiangxi. Pak. Vet. J..

[55] Shafiq M., Zeng M., Permana B., Bilal H., Huang J., Yao F., Jiao X. (2022). Coexistence of *blaNDM–5* and *tet(X4)* in International High-Risk *Escherichia coli* Clone ST648 of Human Origin in China. Front. Microbiol..

[56] Van den Bergh B., Fauvart M., Michiels J. (2017). Formation, Physiology, Ecology, Evolution and Clinical Importance of Bacterial Persisters. FEMS Microbiol. Rev..

[57] Brauner A., Fridman O., Gefen O., Balaban N.Q. (2016). Distinguishing Between Resistance, Tolerance and Persistence to Antibiotic Treatment. Nat. Rev. Microbiol..

[58] Fisher R.A., Gollan B., Helaine S. (2017). Persistent Bacterial Infections and Persister Cells. Nat. Rev. Microbiol..

[59] Balaban N.Q., Helaine S., Lewis K., Ackermann M., Aldridge B., Andersson D.I., Zinkernagel A. (2019). Definitions and Guidelines for Research on Antibiotic Persistence. Nat. Rev. Microbiol..

[60] Ayrapetyan M., Williams T., Oliver J.D. (2018). Relationship Between the Viable but Nonculturable State and Antibiotic Persister Cells. J. Bacteriol..

[61] Bodor A., Bounedjoum N., Vincze G.E., Erdeiné Kis Á., Laczi K., Bende G., Rákhely G. (2020). Challenges of Unculturable Bacteria: Environmental Perspectives. Rev. Environ. Sci. Biotechnol..

[62] Chebotar I.V., Emelyanova M.A., Bocharova J.A., Mayansky N.A., Kopantseva E.E., Mikhailovich V.M. (2021). The Classification of Bacterial Survival Strategies in the Presence of Antimicrobials. Microb. Pathog..

[63] Pangeni B., Bhattarai S., Paudyal H., Chaudhary R.P. (2021). Antibacterial Activity of Selected Ethnomedicinal Plants Popular in Magar Ethnic Community of Palpa District, Western Nepal. Arch. Ecotoxicol..

[64] Neagu R., Popovici V., Ionescu L.E., Ordeanu V., Biță A., Popescu D.M., Gîrd C.E. (2024). Phytochemical screening and antibacterial activity of commercially available essential oils combinations with conventional antibiotics against gram-positive and gram-negative bacteria. Antibiotics.

[65] Gupta V., Kumar R., Chaudhary D., Yadav N. (2016). In-Vitro Analysis of Potential Antibacterial Activity of Three Medicinal Plants. J. Appl. Nat. Sci..

[66] Rauf A., Abu-Izneid T., Rashid U., Alhumaydhi F.A., Bawazeer S., Khalil A.A., Ntsefong G.N. (2020). Anti-Inflammatory, Antibacterial, Toxicological Profile, and In Silico Studies of Dimeric Naphthoquinones from *Diospyros lotus*. BioMed Res. Int..

[67] Mogana R., Teng-Jin K., Wiart C. (2011). In vitro antimicrobial, antioxidant activities and phytochemical analysis of *Canarium patentinervium* Miq. from Malaysia. Biotechnol. Res. Int..

[68] Elbalola A.A., Abbas Z.K. (2023). Phytochemical Diversity, Classification and Antibacterial Activity of Some Medicinal Plant Species from Tabuk (Saudi Arabia). Chem. Biodivers..

[69] Kakaraparthy R., Sruthi K.S., Banerjee M. (2023). Evaluation of Antibacterial and Wound Healing Activities of Ethanolic Extract of *Linum usitatissimum* (Flax Seed) on Rabbits. J. Clin. Pharm. Res..

[70] Shiferaw Z., Sasikumar J.M., Kebede A., Teju E. (2022). Antibacterial Effects of Extracts from *Tanacetum parthenium* L. Leaves. Bact. Emp..

[71] Atta S., Waseem D., Fatima H., Naz I., Rasheed F., Kanwal N. (2023). Antibacterial potential and synergistic interaction between natural polyphenolic extracts and synthetic antibiotic on clinical isolates. Saudi J. Biol. Sci..

[72] Istaufa F., Subagio Y., Suswati I. (2022). *Ricinus communis* L. Leaf Extract as Potential Antibacterial Against the Growth of *Mycobacterium tuberculosis*. Folia Med. Indones..

[73] Puspaningtyas A.R., Nugraha A.S., Retnaningtyas Y., Zulaiha S. (2024). Anti-Tuberculosis Study of *Mycobacterium tuberculosis* H37Rv of *Aspilia pluriceta* Extract and Fractions. Trop. J. Nat. Prod. Res..

[74] Sarkar S., Singh R.P. (2022). *Nyctanthes arbortristis* L.: Perspective of Phytochemical-Based Inhibition of Fatty Acid Biosynthesis in *Mycobacterium tuberculosis*. Int. J. Plant Based Pharm..

[75] Moni E.D.F.N., Betote P.H.D., Kom C.W., Benga C.F.M., Tchamgoue A.D., Nyegue M.A. (2021). Inhibitory Effects of Hydroethanolic Extracts from Three Cameroonian Medicinal Plants on Proteins Inflammation and Growth of Multi-Resistant Strains of *Mycobacterium tuberculosis*. J. Drug Deliv. Ther..

[76] Issa N.A. (2024). Evaluation the Antimicrobial Activity of Essential Oils Against Veterinary Pathogens, Multidrug-resistant Bacteria and Dermatophytes. Pak. Vet. J..

[77] Aljohani A.S.M. (2023). Botanical compounds: A promising approach to control *Mycobacterium* species of veterinary and zoonotic importance. Pak. Vet. J..

[78] Qian W., Liu M., Fu Y., Zhang J., Liu W., Li J., Li X., Li Y., Wang T. (2020). Antimicrobial mechanism of luteolin against *Staphylococcus aureus* and *Listeria monocytogenes* and its antibiofilm properties. Microb. Pathog..

[79] Zhong Z.X., Zhou S., Liang Y.J., Wei Y.Y., Li Y., Long T.F., Sun J. (2023). Natural flavonoids disrupt bacterial iron homeostasis to potentiate colistin efficacy. Sci. Adv..

[80] Osagie E., Erhauyi O., Udogadi N., Olalekan S. (2021). Action of Fractionated *Moringa oleifera* Lam Leaf Extracts on Multidrug Resistant *Pseudomonas aeruginosa* Strains. Int. J. Clin. Exp. Med. Res..

[81] Musuasua M., Kabena O., Kalanda L., Kangudia B., Mutembue D., Masens D., Mpiana P. (2022). Phytochemical Screening and In Vitro Antibacterial Activity of Aqueous Extracts of *Phyllanthus muellerianus* (Kuntze) Exell from Kasaï Oriental (DRC) on a Few Bacterial Strains. Int. J. Pathog. Res..

[82] Semwal P., Painuli S., Badoni H., Bacheti R. (2018). Screening of Phytoconstituents and Antibacterial Activity of Leaves and Bark of *Quercus leucotrichophora* A. Camus from Uttarakhand Himalaya. Clin. Phytosci..

[83] Silva Leandro M.K.D.N., Rocha J.E., Bezerra C.F., Freitas P.R., Feitosa J.H.F., Bezerra V.B., Barros R.d.O., Leandro L.M.G., Aguiar J.J.d.S., Pereira P.S. (2020). Modulation of Antibiotic Resistance by the Essential Oil of *Ocimum gratissimum* L. in Association with Light-Emitting Diodes (LED) Lights. Z. Naturforsch. C.

[84] Kumari B., Raveesha H. (2024). Phytochemical Analysis and Antibacterial Activity of *Andrographis lineata* Nees (Acanthaceae). Curr. Bot..

[85] Mambe F., Na-Iya J., Fotso G., Ashu F., Ngameni B., Ngadjui B., Kuete V. (2019). Antibacterial and Antibiotic Modifying Potential of Crude Extracts, Fractions, and Compounds from *Acacia polyacantha* Willd. Against MDR Gram-Negative Bacteria. Evid. Based Complement. Alternat. Med..

[86] Gangwar B., Kumar S., Darokar M. (2023). Antioxidant Phytochemicals as Novel Therapeutic Strategies Against Drug-Resistant Bacteria.

[87] Borges A., Abreu A., Dias C., Saavedra M., Borges F., Simões M. (2016). New Perspectives on the Use of Phytochemicals as an Emergent Strategy to Control Bacterial Infections Including Biofilms. Molecules.

[88] Barati M., Chahardehi A.M., Barati M., Chahardehi A.M. (2023). Alkaloids: The Potential of Their Antimicrobial Activities of Medicinal Plants. Medicinal Plants—Chemical, Biochemical, and Pharmacological Approaches.

[89] Parai D., Banerjee M., Dey P., Mukherjee S.K. (2020). Reserpine Attenuates Biofilm Formation and Virulence of *Staphylococcus aureus*. Microb. Pathog..

[90] Saha S., Do T., Maycock J., Wood S., Boesch C. (2023). Antibiofilm Efficacies of Flavonoid-Rich Sweet Orange Waste Extract Against Dual-Species Biofilms. Pathogens.

[91] Faleye O.S., Lee J.-H., Lee J. (2023). Selected Flavonoids Exhibit Antibiofilm and Antibacterial Effects Against *Vibrio* by Disrupting Membrane Integrity, Virulence and Metabolic Activities. Biofilm.

[92] Raorane C.J., Lee J.-H., Kim Y.-G., Rajasekharan S.K., García-Contreras R., Lee J. (2019). Antibiofilm and Antivirulence Efficacies of Flavonoids and Curcumin Against *Acinetobacter baumannii*. Front. Microbiol..

[93] Shang F., Wang H., Xue T. (2020). Anti-Biofilm Effect of Tea Saponin on a Streptococcus agalactiae Strain Isolated from Bovine Mastitis. Animals.

[94] Li L., Wei M., Yu H., Xie Y., Guo Y., Cheng Y., Yao W. (2023). Antifungal Activity of *Sapindus* Saponins Against *Candida albicans*: Interruption of Biofilm Formation. J. Herb. Med..

[95] Fink R., Filip S. (2023). Surface-Active Natural Saponins: Properties, Safety, and Efficacy. Int. J. Environ. Health Res..

[96] Adnan M., Siddiqui A.J., Ashraf S.A., Ashraf M.S., Alomrani S.O., Alreshidi M., Tepe B., Sachidanandan M., Danciu C., Patel M. (2023). Saponin-Derived Silver Nanoparticles from *Phoenix dactylifera* (Ajwa Dates) Exhibit Broad-Spectrum Bioactivities Combating Bacterial Infections. Antibiotics.

[97] Villanueva X., Zhen L., Ares J.N., Vackier T., Lange H., Crestini C., Steenackers H.P. (2023). Effect of Chemical Modifications of Tannins on Their Antimicrobial and Antibiofilm Effect Against Gram-Negative and Gram-Positive Bacteria. Front. Microbiol..

[98] Qin J., Yu L., Peng F., Ye X., Li G., Sun C., Cheng F., Peng C., Xie X. (2023). Tannin Extracted from *Penthorum chinense* Pursh, a Potential Drug with Antimicrobial and Antibiofilm Effects Against Methicillin-Sensitive *Staphylococcus aureus* and Methicillin-Resistant *Staphylococcus aureus*. Front. Microbiol..

[99] Alejo-Armijo A., Cobo A., Alejo-Armijo A., Altarejos J., Salido S., Ortega-Morente E. (2025). Evaluation of Antibacterial and Antibiofilm Properties of Phenolics with Coumarin, Naphthoquinone and Pyranone Moieties Against Foodborne Microorganisms. Molecules.

[100] Glamočlija J., Ćirić A., Nikolić M., Fernandes Â., Barros L., Calhelha R.C., Ferreira I.C.F.R., Soković M., van Griensven L.J.L.D. (2015). Chemical Characterization and Biological Activity of Chaga (*Inonotus obliquus*), a Medicinal “Mushroom”. J. Ethnopharmacol..

[101] Slobodníková L., Fialová S., Rendeková K., Kováč J., Mučaji P. (2016). Antibiofilm Activity of Plant Polyphenols. Molecules.

[102] Fydrych D., Jeziurska J., Wełna J., Kwiecińska-Piróg J. (2025). Potential Use of Selected Natural Compounds with Anti-Biofilm Activity. Int. J. Mol. Sci..

[103] Zhang C., Li Z., Pan Q., Fan L., Pan T., Zhu F., Pan Q., Shan L., Zhao L. (2022). Berberine at Sub-Inhibitory Concentration Inhibits Biofilm Dispersal in *Staphylococcus aureus*. Microbiology.

[104] Fu Y., Liu W., Liu M., Zhang J., Yang M., Wang T., Qian W. (2021). In vitro anti-biofilm efficacy of sanguinarine against carbapenem-resistant *Serratia marcescens*. Biofouling.

[105] Das S., Malik M., Dastidar D.G., Roy R., Paul P., Sarkar S., Chakraborty P., Maity A., Dasgupta M., Tribedi P. (2024). Piperine, a phytochemical prevents the biofilm city of methicillin-resistant *Staphylococcus aureus*: A biochemical approach to understand the underlying mechanism. Microb. Pathog..

[106] Matilla-Cuenca L., Gil C., Cuesta S., Rapún-Araiz B., Žiemytė M., Mira A., Lasa I., Valle J. (2020). Antibiofilm Activity of Flavonoids on Staphylococcal Biofilms through Targeting BAP Amyloids. Sci. Rep..

[107] Zhang Y., Zhang Y., Ma R., Sun W., Ji Z. (2023). Antibacterial Activity of Epigallocatechin Gallate (EGCG) Against *Shigella flexneri*. Int. J. Environ. Res. Public Health.

[108] Paluch E., Bortkiewicz O., Widelski J., Duda-Madej A., Gleńsk M., Nawrot U., Lamch Ł., Długowska D., Sobieszczańska B., Wilk K.A. (2024). A Combination of β-Aescin and Newly Synthesized Alkylamidobetaines as Modern Components Eradicating the Biofilms of Multidrug-Resistant Clinical Strains of *Candida glabrata*. Int. J. Mol. Sci..

[109] Antolak H., Mizerska U., Berłowska J., Otlewska A., Kręgiel D. (2018). *Quillaja saponaria* Saponins with Potential to Enhance the Effectiveness of Disinfection Processes in the Beverage Industry. Appl. Sci..

[110] Ratti A., Fassi E.M.A., Forlani F., Mori M., Villa F., Cappitelli F., Sgrignani J., Roda G., Cavalli A., Villa S. (2023). Mechanistic Insights into the Antibiofilm Mode of Action of Ellagic Acid. Pharmaceutics.

[111] Ulrey R.K., Barksdale S.M., Zhou W., van Hoek M.L. (2014). Cranberry Proanthocyanidins Have Anti-Biofilm Properties Against *Pseudomonas aeruginosa*. BMC Complement. Altern. Med..

[112] Qin T., Chen K., Xi B., Pan L., Xie J., Lu L., Liu K. (2023). In Vitro Antibiofilm Activity of Resveratrol Against *Aeromonas hydrophila*. Antibiotics.

[113] Asadi S., Nayeri-Fasaei B., Zahraei-Salehi T., Yahya-Rayat R., Shams N., Sharifi A. (2023). Antibacterial and Anti-Biofilm Properties of Carvacrol Alone and in Combination with Cefixime Against *Escherichia coli*. BMC Microbiol..

[114] Bisso Ndezo B., Tokam Kuaté C.R., Dzoyem J.P. (2021). Synergistic Antibiofilm Efficacy of Thymol and Piperine in Combination with Three Aminoglycoside Antibiotics Against *Klebsiella pneumoniae* Biofilms. Can. J. Infect. Dis. Med. Microbiol..

[115] Albano C., Nabawy A., Tran W.C., Prithviraj M., Kado T., Hassan M.A., Makabenta J.M.V., Rotello V.M., Morita Y.S., Neyrolles O. (2025). Effective killing of *Mycobacterium abscessus* biofilm by nanoemulsion delivery of plant phytochemicals. Microbiology Spectrum..

[116] Iskandar K., Murugaiyan J., Hammoudi Halat D., Hage S.E., Chibabhai V., Adukkadukkam S., Roques C., Molinier L., Salameh P., Van Dongen M. (2022). Antibiotic Discovery and Resistance: The Chase and the Race. Antibiotics.

[117] Halawa E.M., Fadel M., Al-Rabia M.W., Behairy A., Nouh N.A., Abdo M., Olga R., Fericean L., Atwa A.M., El-Nablaway M. (2024). Antibiotic Action and Resistance: Updated Review of Mechanisms, Spread, Influencing Factors, and Alternative Approaches for Combating Resistance. Front. Pharmacol..

[118] Manner S., Fallarero A. (2018). Screening of Natural Product Derivatives Identifies Two Structurally Related Flavonoids as Potent Quorum Sensing Inhibitors Against Gram-Negative Bacteria. Int. J. Mol. Sci..

[119] Sikdar R., Elias M. (2020). Quorum Quenching Enzymes and Their Effects on Virulence, Biofilm and Microbiomes: A Review of Recent Advances. Expert Rev. Anti Infect. Ther..

[120] Rehman Z.U., Momin A.A., Aldehaiman A., Irum T., Grünberg R., Arold S.T. (2022). The Exceptionally Efficient Quorum Quenching Enzyme LrsL Suppresses *Pseudomonas aeruginosa* Biofilm Production. Front. Microbiol..

[121] Fong J., Zhang C., Yang R., Boo Z.Z., Tan S.K., Nielsen T.E., Givskov M., Liu X.-W., Bin W., Su H. (2018). Combination Therapy Strategy of Quorum Quenching Enzyme and Quorum Sensing Inhibitor in Suppressing Multiple Quorum Sensing Pathways of *P. aeruginosa*. Sci. Rep..

[122] Naga N.G., Zaki A.A., El-Badan D.E., Rateb H.S., Ghanem K.M., Shaaban M.I. (2023). Inhibition of *Pseudomonas aeruginosa* Quorum Sensing by Methyl Gallate from *Mangifera indica*. Sci. Rep..

[123] Hetta H.F., Ramadan Y.N., Rashed Z.I., Alharbi A.A., Alsharef S., Alkindy T.T., Alkhamali A., Albalawi A.S., Battah B., Donadu M.G. (2024). Quorum Sensing Inhibitors: An Alternative Strategy to Win the Battle Against Multidrug-Resistant (MDR) Bacteria. Molecules..

[124] Mitropoulou G., Karapantzou I., Tsimogiannis D., Oreopoulou V., Lazăr V., Kourkoutas Y. (2025). Inhibitory Effects of Essential Oils and Extracts of the Water-Steam Distillation Residues from Greek Herbs on Adherent Biofilm Formation by Common Pathogens. Appl. Sci..

[125] Chimi L.Y., Bisso B.N., Njateng G.S.S., Dzoyem J.P. (2023). Antibiotic-Potentiating Effect of Some Bioactive Natural Products Against Planktonic Cells, Biofilms, and Virulence Factors of *Pseudomonas aeruginosa*. BioMed. Res. Int..

[126] Getahun M., Nesru Y., Ahmed M., Satapathy S., Shenkute K., Gupta N., Naimuddin M. (2023). Phytochemical Composition, Antioxidant, Antimicrobial, Antibiofilm, and Antiquorum Sensing Potential of Methaol Extract and Essential Oil from *Acanthus polystachyus* Delile (Acanthaceae). ACS Omega.

[127] Nikolic I., Aleksic Sabo V., Gavric D., Knezevic P. (2024). Anti-*Staphylococcus aureus* Activity of Volatile Phytochemicals and Their Combinations with Conventional Antibiotics Against Methicillin-Susceptible *S. aureus* (MSSA) and Methicillin-Resistant *S. aureus* (MRSA) Strains. Antibiotics.

[128] Boriollo M.F.G., Marques M.B., da Silva T.A., Da Silva J.J., Dias R.A., Silva Filho T.H.N., Melo I.L.R., dos Santos Dias C.T., Bernardo W.L.D.C., de Mello Silva Oliveira N. (2020). Antimicrobial potential, phytochemical profile, cytotoxic and genotoxic screening of *Sedum praealtum* A. DC. (balsam). BMC Complement. Med. Ther..

[129] Anywar G.U., Kakudidi E., Oryem-Origa H., Schubert A., Jassoy C. (2022). Cytotoxicity of Medicinal Plant Species Used by Traditional Healers in Treating People Suffering from HIV/AIDS in Uganda. Front. Toxicol..

[130] Sun Y., Mao W., Cao J., Hao Pgao Jianguo S., Yin K., Gu K., Zhao H. (2024). Chinese Medicine Monomers Inhibit Biofilm Formation in Multidrug-Resistant *Pasteurella multocida* Isolated from Cattle Respiratory Infections. Pak. Vet. J..

[131] Alves-Barroco C., Botelho A.M.N., Américo M.A., Fracalanzza S.E.L., de Matos A.P.A., Guimaraes M.A., Ferreira-Carvalho B.T., Figueiredo A.M.S., Fernandes A.R. (2022). Assessing in vivo and in vitro biofilm development by *Streptococcus dysgalactiae* subsp. Dysgalactiae using a murine model of catheter-associated biofilm and human keratinocyte cell. Front. Cell. Infect. Microbiol..

[132] Santhosh S.K., Sarojini S. (2025). Antibiofilm and antiquorum properties of ethanolic leaf extracts of *Syzygium jambos* and *Psidium guajava* and their gel formulation for wound healing applications. Plant Sci. Today.

